# Sex-specific association of a common *GNAS* polymorphism with self-reported cognitive empathy in healthy volunteers

**DOI:** 10.1371/journal.pone.0206114

**Published:** 2018-10-26

**Authors:** Franz Korbinian Huetter, Peter Alexander Horn, Winfried Siffert

**Affiliations:** 1 Institut für Pharmakogenetik, Universitätsklinikum Essen, Universität Duisburg Essen, Essen, Germany; 2 Institut für Transfusionsmedizin, Universitätsklinikum Essen, Universität Duisburg Essen, Essen, Germany; Public Library of Science, UNITED KINGDOM

## Abstract

**Background:**

In a recent study, we found associations of a common oxytocin receptor *(OXTR)* polymorphism with inter-individual differences in empathy, especially with emotional empathy in women. Many other studies found specific associations of oxytocin, arginine-vasopressin, serotonin and dopamine receptor gene polymorphisms with various aspects of trait empathy. As all these receptors belong to the guanine-binding protein (G protein) coupled receptor family, it is a reasonable assumption, that alterations in genes encoding G protein subunits also influence the signal transduction in empathy related circuits. However, to the best of our knowledge, these genomic variations have not yet been studied in genetic research on empathy.

**Methods:**

Here, we analysed associations of a common polymorphism of the *GNAS* gene (C393T) in a previously characterized sample of 421 healthy blood donors (231 M, 190 F; age 18–74). The *GNAS* gene encodes the G protein adenylyl cyclase stimulator (Gαs) G protein subunit, which activates cyclic adenosine monophosphate (cAMP)-dependent pathways by stimulating the adenylyl cyclase. Cognitive and emotional aspects of dispositional empathy were tested using Davis’ Interpersonal Reactivity Index (IRI).

**Results:**

In the complete sample, associations of C393T genotype with IRI empathy scores, including cognitive empathy (p = 0.055) and perspective taking (p = 0.057) scores did not reach a level of significance. None of the IRI scores was near to being significantly associated with C393T genotype for men alone. In females, however, genotype was significantly associated with cognitive empathy (r = -.204, p = 0.005) and perspective taking (r = -.209, p = 0.004), accounting for 4.2% and 4.4% of variability. The association of genotype with perspective taking remained significant after adjustment for multiple comparisons (p = 0.045). The 393C-allele, which had been identified as a risk factor in several medical conditions such as hypertension, obesity and diabetes, was associated with higher cognitive empathy compared to the T allele in our sample.

**Conclusions:**

The results suggest a significant association of *GNAS* C393T genotypes with the cognitive empathic capacity of perspective taking. This association could only be found in female participants.

## Introduction

Empathy is a complex psychological capacity including both a cognitive appraisal of other people’s experience and an affective reaction to their emotions, which may or may not result in a behavioural outcome such as offering help to others in need [1]. While disease-specific empathic deficits have been identified in several conditions like autism [2], schizophrenia [3], psychopathy [4] and depression [5], psychological research has established a considerable variability of empathic dispositions in healthy subjects [6]. One of the most frequently used psychological tests of such empathic dispositions is Davis’ Interpersonal Reactivity Index (IRI) [7,8], a well validated multidimensional self-report measure of four aspects of cognitive and affective empathy.

As opposed to situationally dependent empathic states, stable empathic traits of personality as those measured by the IRI have been associated with underlying neuronal endophenotypes emerging as a result of environmental as well as genetic influences [9]. Whereas neuronal substrates of the four IRI scales have been suggested [10] and distinct brain circuits for cognitive and emotional empathic reactions are supported by a growing body of evidence [11], small effect sizes for polymorphisms of major candidate genes [12] suggest that there are still many white areas on the map of genetic influences on empathic disposition.

We recently showed that a common oxytocin receptor (*OXTR*) polymorphism (rs53576) is associated with emotional aspects of empathy in a large cohort of 421 healthy blood donors [13]. Interestingly, this effect was restricted to the female group, but weak or absent in male study participants in our sample. Effects of the polymorphism accounted for a maximum of 2.1% of the variability in female IRI scores (effect size r = .146, p = 0.04) and were thus only slightly larger than those observed in a recent meta-analysis (n = 4955) on the association of this polymorphism with general sociality (Cohen’s d = 0.11, p = .02) [14]. For this reason, we discussed our results in the context of a polygenetic determination of empathic dispositions. In fact, a considerable number of genetic variations have been associated with empathic dispositions. Among these are several *OXTR* polymorphisms [15], variations in the *CD38* gene, which codes for a transmembrane protein engaged in the secretion of oxytocin [16–18] polymorphisms of arginine-vasopressin receptors [19,20] and of several genes regulating monoaminergic, especially serotonergic and dopaminergic activity [17,21,22]. While these polymorphisms, along with gene by environment interactions [23,24] are likely to add up to cumulative genotype effects on empathic disposition, a common denominator that would combine the plethora of individual empathy-related variations to a bigger picture is still missing.

In the present study, we hypothesized that such a common denominator may be found in the fact that all of the above mentioned modulator systems rely on G-protein coupled signal transduction pathways. Like many other serpentine receptors, the receptors of the nonapeptides oxytocin and arginine-vasopressin as well as the serotonin and dopamine receptors transmit their signals through activation of heterotrimeric G proteins. These heterotrimers are ubiquitously expressed and thus have a considerable influence on the state of the complete organism. As empathic responses have been previously associated with the activation of a wide range of distributed brain circuits [10] and of the autonomic nervous system [25], contributing to automatic embodied-simulation (ES) via visceromotor efferences and viscerosensitive afferences [11], studying variations in such omnipresent molecular agents is alluring in the context of empathy research. Heterotrimers consist of alpha-, beta- and gamma subunits and their function and tissue-specific expression are fairly well understood. The Gαs subunit which couples many receptors to the adenylyl cyclase-cAMP system, including the arginine vasopressin (AVP) receptor 2, the 5-hydoxytryptophan (5-HT) 4 and 7 receptors and dopamine receptors of the D1-like family, is encoded by the *GNAS* gene located on chromosome 20q13.32. The *GNAS* locus has a highly complex expression pattern due to imprinting and gives rise to a variety of *GNAS* products.

In order to define potential genetic polymorphisms in the *GNAS* gene which could increase the risk for essential hypertension, Jia et al. [26] detected a silent C393T polymorphism (rs7121). Subsequently, this polymorphism was associated with a variety of pathological states [27] and with outcome of patients with cancer [28]. Although this nucleotide exchange leaves the amino acid composition of Gαs unaffected, it may nevertheless impact upon Gαs expression. Frey et al. [29] suggested that this nucleotide exchange may alter the structure of the pre-mRNA, thereby potentially also affecting mRNA stability. This assumption was supported by the fact, that Gαs mRNA expression was indeed highest in bladder cancer, adipose tissue, and heart tissue from probands with 393 TT genotypes.

Here we investigated potential associations of *GNAS* C393T genotypes with different aspects of human empathy using DNA from a large sample of previously characterized healthy individuals [13].

## Materials and methods

### Ethics statement

We obtained approval of the study from the ethics committee of the Medical Faculty of the University of Duisburg-Essen (reference number 14-5797-BO). All participants gave written informed consent before completing the questionnaire and donating an additional blood sample required for this study. We acquired all data anonymously and limited demographic data to sex and age.

### Participants, data and sample acquisition

500 blood donors were initially recruited at the Essen University Hospital, Institute for Transfusion Medicine, in 2015. Details have been reported previously [13].

Prior to evaluating the results, we excluded all questionnaires with ambiguous or incomplete answers or without matching blood samples, which reduced the number of evaluated individuals to 421.

The remaining 190 female and 231 male participants were between 18 and 74 years of age (*M* 36.6; *median* 30). Men were between 18 and 74 years (*M* 38.9; *median* 35) and women were between 18 and 73 years of age (*M* 33.7, *median* 25).

### The interpersonal reactivity index

Participants answered the 28 items of Davis’ Interpersonal Reactivity Index (IRI) in German language [30]. The IRI measures the following four dimensions of empathic traits [7,8]:

1. Perspective taking (PT) is conceived of as an inclination to see a situation from another person’s viewpoint and is, therefore, akin to the concept of Theory-of-Mind [31].
2. Fantasy (FT) is defined as the disposition of an individual to easily identify with characters in fictional genres such as novels and movies.
3. The empathic concern (EC) scale measures the degree of self-reported compassion and care for other people in negative situations. This other-oriented aspect of empathy has also been shown to be associated with altruistic actions in favour of others in need [32,33].
4. The personal distress (PD) scale refers to a tendency towards a stressful and potentially avoidant reaction to perceiving other persons in unfortunate situations.

In the validation sample of the German version of the IRI, the original IRI factor structure was confirmed and comparable measures of reliability (Cronbach’s α = .78), selectivity and external validity were found [34].

### Determination of the *GNAS* C393T genotypes

Isolation of genomic DNA from blood was performed using a commercially available kit (QIAamp DNA Blood Mini Kit, Qiagen, Hilden, Germany) following the manufacturer’s instructions. Genotypes of the C393T polymorphism were determined by PCR using the following primers: forward primer, 5`- TGT GGC CGC CAT GAG CAA -3`and reverse primer, 5`- TAA GGC CAC ACA AGT CGG GGT -3`. After an initial denaturation at 95°C, 38 cycles of DNA amplification were done using Ampliqon Mastermix (Odense M, Denmark) at 95°C for 30s, 64°C for 30s, 72°C for 30s and a final elongation step of 72°C for 10 min. The 145-bp PCR products were digested using the restriction enzyme BSE GI (Thermo Fisher Scientific, Waltham, MA USA) and analysed on a 2.8% agarose gel. The unrestricted products (145-bp) represent the TT genotype, the completely restricted products (77bp) represent the CC genotype.

### Statistics

In line with the prevailing practice in IRI-based research, we classified PT and FT as cognitive and EC and PD as emotional aspects of empathy [2,15] and calculated cumulative scores [15,35,36] as follows:

1. trait empathy score (TES): TES = PT+FT+EC+PD
2. cognitive empathy score (CES): CES = PT+FT
3. emotional empathy score (EES): EES = EC+PD

We calculated descriptive measures for all individual and cumulative IRI scores. As a Kolmogorov-Smirnov test with Lillefors significance correction revealed that the majority of scores were not normally distributed, we applied non-parametric statistics without exception. Our recent research [13] had shown an allele-dosage effect of an oxytocin receptor polymorphism on certain IRI scores. A similar allele-dosage effect has been previously shown for the *GNAS* C393T polymorphism in other contexts, with the C-allele representing a risk factor for a number of medical conditions [27,29]. We, therefore, hypothesized C393T genotypes to represent ordered alternatives from CC to TC to TT. We applied the Jonckheere-Terpstra-Test to test for associations between empathy scores and genotype. This test has been identified as the most appropriate non-parametric hypothesis test for genotypes with ordered alternatives [37]. Possible associations between age and genotype and between sex and genotype were tested using contingency tables and Pearson’s Chi-squared tests. Spearman’s rank correlation coefficients were calculated as non-parametic measures of effect size of genotypes on IRI scores. Consistent with the non-parametric approach, location and dispersion were reported using medians and interquartile ranges of empathy scores instead of mean values and standard deviations. Possible deviations from the Hardy-Weinberg equilibrium were calculated with a public domain software [38]. Allele frequencies and genotype distributions were compared to a published sample of 820 healthy Caucasian blood donors [29]. Power and sample size analyses were performed using the public domain software G*Power 3.1 [39]. Differences were regarded significant at p<0.05 for the complete sample. Adjustment for multiple comparisons was made using the Benjamini-Hochberg method [40]. Statistical analyses were done using SPSS 23.0.

## Results

### Distribution of genotypes

The distribution of the C393T genotypes was in accordance with the Hardy-Weinberg equilibrium for the complete sample as well as for males and females alone. T-allele frequency (48%) as well as genotype distribution (CC, n = 124, 29.5%; TC, n = 189, 44.9%; TT, n = 108, 25.7%) were not significantly different from a published genotype distribution of 820 healthy Caucasian blood donors (T-allele frequency 47%; CC, n = 235, 26.7%; TC, n = 403, 49,2%; TT, n = 182, 22,2%) [29], based on a calculation of differences and comparative error. Chi-squared tests revealed no significant associations between allele frequencies and sex (p = 0.8) or age (p = 0.5).

### Genotype and empathy scores

As shown in Table 1, none of the IRI scores, including the cognitive empathy score (CES: p = 0.055) and the perspective taking score (PT: p = 0.057) were significantly associated with C393T genotype.

**Table 1:** Empathy scores by *GNAS* (C393T) genotype for all participants.

	All		CC		TC		TT		p*
	(n = 421 | 100%)		(n = 124 | 30%)		(n = 189 | 44.9%)		(n = 108 | 25.1%)		
	Mdn	IQR	Mdn	IQR	Mdn	IQR	Mdn	IQR	
**TES**	60	14 (53–67)	60	14 (54–68)	60	14 (52–67)	59.5	12 (53–65)	0.24
**CES**	32	9 (27–36)	32	10 (28–38)	32	9 (28–36)	30	8 (27–35)	0.06
**PT**	18	5 (15–20)	18	5 (15–20)	18	5 (15–20)	17	5 (14–19)	0.06
**FT**	14	6 (11–17)	15	7 (11–18)	14	7 (11–18)	14	7 (10–17)	0.14
**EES**	28	8 (24–32)	29	7 (25–32)	28	7 (24–31)	28	9 (24–33)	0.50
**EC**	17	4 (15–19)	17	3 (16–19)	17	4 (15–19)	17	4 (15–19)	0.91
**PD**	11	6 (8–14)	12	6 (8–14)	11	6 (8–14)	11	6 (8–14)	0.63

TES: trait empathy score, CES: cognitive empathy score, PT: perspective taking; FT: fantasy; EES: emotional empathy score. EC: empathic concern; PD: personal distress. Mdn: medians were included to show ranked effects. IQR: interquartile range was used as a measure of dispersion, with the 25th and the 75th percentiles in brackets (values with decimal places were rounded to whole numbers for reasons of space).

* *p* values were calculated using the Jonckheere-Terpstra-Test.

### Association of genotype and empathy by sex

Due to the sex differences observed in our previous study [13] and in other studies relating genotypes to empathy [41–44], we conducted an exploratory analysis into males and females separately, to test for a possible sexually dimorphic impact of the C393T genotype on empathy. Of note, none of the IRI scores was near to being significantly associated with C393T genotypes for men (see Table 2).

**Table 2:** Empathy scores by *GNAS* (C393T) genotype for male participants.

	All		CC		TC		TT		p*
	(n = 231 | 100%)		(n = 66 | 28.6%)		(n = 107 | 46.3%)		(n = 58 | 25.1%)		
	Mdn	IQR	Mdn	IQR	Mdn	IQR	Mdn	IQR	
**TES**	56	12 (49–61)	56	13 (49–62)	56	13 (50–63)	57	13 (48–61)	0.78
**CES**	30	8 (26–34)	30	7 (26–33)	30	8 (26–34)	30	8 (26–34)	0.97
**PT**	17	6 (14–20)	17	6 (14–20)	17	5 (15–20)	17	5 (15–20)	0.98
**FT**	13	6 (10–16)	13	7 (10–17)	13	5 (11–16)	13	7 (9–16)	0.79
**EES**	26	7 (22–29)	26	6 (24–30)	26	6 (23–29)	25.5	6 (22–28)	0.24
**EC**	16	4 (14–18)	16	4 (14–18)	16	4 (14–18)	16	5 (13–18)	0.48
**PD**	9	5 (7–12)	11	6 (7–13)	9	5 (7–12)	9	6 (7–13)	0.39

TES: trait empathy score, CES: cognitive empathy score, PT: perspective taking; FT: fantasy; EES: emotional empathy score. EC: empathic concern; PD: personal distress. Mdn: medians were included to show ranked effects. IQR: interquartile range was used as a measure of dispersion, with the 25th and the 75th percentiles in brackets (values with decimal places were rounded to whole numbers for reasons of space).

* *p* values were calculated using the Jonckheere-Terpstra-Test.

In females (see Table 3), the cognitive empathy score (CES) and the perspective taking [PT] score showed nominally significant associations with *GNAS* (C393T) genotypes (CES: p = 0.005 and PT: p = 0.004). Median scores of cognitive empathy and perspective taking decreased from CC to TT genotypes for CES (median score ± 1/2 IQR: CC 35.5 ± 3.5, TC 34.5 ± 4.5, TT 32.5 ± 3.5) and between C-allele carriers and T homozygotes for PT (median PT score ± ± 1/2 IQR: CC 18 ± 2.5, TC 18 ± 3, TT 17 ± 2). A calculation of the grouped medians for PT also revealed an allele-dosage effect within the group of C-allele carriers, with grouped median PT scores of 18.4 for CC, 18.2 for TC and 16.6 for TT genotypes respectively. Consistently, Spearman’s rank correlation analysis showed negative correlations of CES and PT with genotype. Effect sizes of genotype on CES were r = -.204, p = 0.005, 95% CI [-0.294, -0.111]. Genotype accounted for 4.2% of the variability in female cognitive empathy ranks. Effect sizes of genotype on PT were r = -.209, p = 0.004, 95% CI [-0.299, -0.116]. Genotype accounted for 4.4% of the variability in female perspective taking ranks.

**Table 3:** Empathy scores by *GNAS* (C393T) genotype for female participants.

	All		CC		TC		TT		p*
	(n = 190 | 100%)		(n = 58 | 30.5%)		(n = 82 | 43.2%)		(n = 50 | 26.3%)		
	Mdn	IQR	Mdn	IQR	Mdn	IQR	Mdn	IQR	
**TES**	65	13 (58–71)	65	12 (60–72)	66	13 (58–71)	64	13 (57–70)	0.14
**CES**	34	8 (30–38)	35.5	7 (32–39)	34.5	9 (30–39)	32.5	7 (28–35)	0.01
**PT**	18	5 (15–20)	18	5 (16–21)	18	6 (15–21)	17	4 (14–18)	<0.001
**FT**	16	6 (13–19)	17	6 (14–20)	17	7 (13–20)	15	6 (12–18)	0.07
**EES**	31	8 (27–35)	31	7 (28–35)	30	7 (27–34)	31.5	9 (27–36)	0.62
**EC**	18	4 (16–20)	18	3 (17–20)	17	4 (16–20)	18.5	3 (17–20)	0.50
**PD**	13	5 (13–15)	13	5 (10–15)	13	4 (11–15)	13	6 (10–16)	0.65

TES: trait empathy score, CES: cognitive empathy score, PT: perspective taking; FT: fantasy; EES: emotional empathy score. EC: empathic concern; PD: personal distress. Mdn: medians were included to show ranked effects. IQR: interquartile range was used as a measure of dispersion, with the 25th and the 75th percentiles in brackets (values with decimal places were rounded to whole numbers for reasons of space).

* *p* values were calculated using the Jonckheere-Terpstra-Test.

## Adjustment for multiple comparisons

As we did not find a significant association of the *GNAS* C393T polymorphism with empathy scores in the complete sample, post hoc testing for sex-specific effects, though guided by our assumptions based on previous research [13,41–44], had to take into account the problem of multiple comparisons.

Choosing a conservative approach, we adjusted for comparing the four IRI scores perspective taking (PT), fantasy (FT), empathic concern (EC) and personal distress (PD) in three groups (in the complete sample, in males and in females), resulting in an adjustment for 12 comparisons. The trait empathy score (TES), the cognitive empathy score (CES) and the emotional empathy score (EES) were not adjusted for, because they are calculated sum scores of the other IRI scores and do not constitute distinct measurements of empathy. We further adjusted for a previous association of the same sample with an oxytocin receptor (OXTR) gene polymorphism [13]. As OXTR genotype was significantly associated with empathy in the complete sample in this previous study, all further analyses were strictly exploratory, so that a single adjustment was made. In total, adjustment was, made for 13 p-values using the Benjamini-Hochberg method. Perspective taking in the female group (p = 0.004) remained significant with a Benjamini-Hochberg adjusted p-value of p = 0.049. As the nominally significant female CES score (p = 0.01) contains the PT score and the FT score, which was not significantly associated with GNAS C393T genotype (p = 0.07), the association with the CES score cannot be assumed to remain significant after adjustment for multiple comparisons.

## Analysis of statistical power

Given the sample size of n = 421 in the complete group, n = 231 in the male subgroup and n = 190 in the female subgroup, we calculated the minimum effect size detectable with 80% power at an alpha level of p = 0.05. The critical effect size was calculated to be r = ± 0.10 for the complete sample, r = ± 0.13 for the male sample and r = ± 0.14 for the female sample. The detected correlations of genotype in the female group were r = -.204 for CES and r = -.209 for PT.

## Discussion

### Association of cognitive empathy with C393T genotype

**Fig 1:**
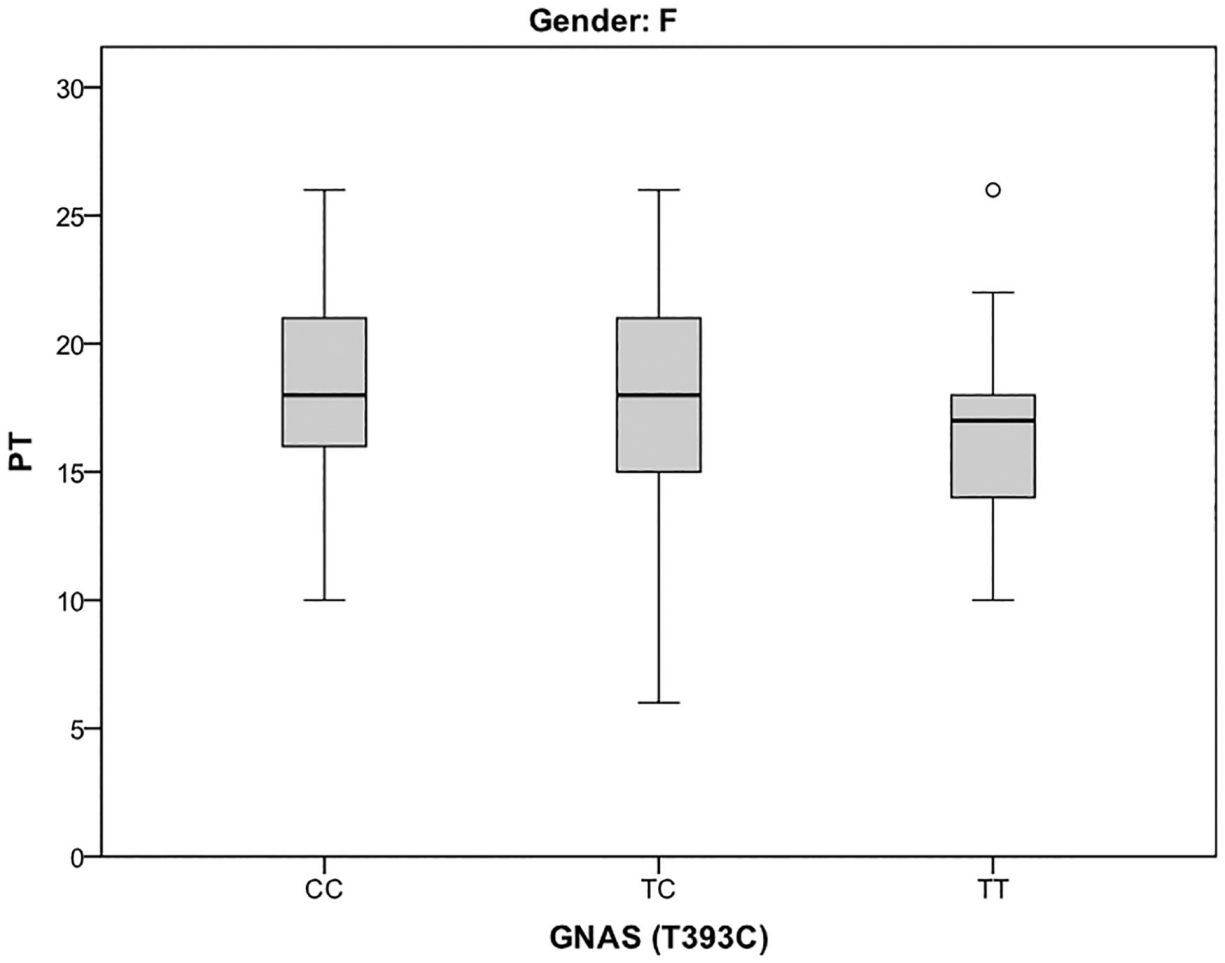
Perspective taking [PT] scores of female participants across genotypes. Boxes represent the 25th percentile, the median and the 75th percentile. Whiskers represent the minima and maxima of PT scores excluding outliers.

Our study shows a significant association of *GNAS* C393T genotypes with IRI perspective taking (PT) scores in female participants (see Fig 1 for perspective taking).

To the best of our knowledge, this is the first study investigating an association of this polymorphism with dispositional empathy or other personality traits. While this variation has been associated with several medical conditions or identified as a predictor of outcome in various malignancies [27–29], we are aware of only one study which has previously observed C393T effects on psychological functions. Minoretti et al. [45] found a significantly increased 393T-allele frequency in schizophrenic patients with negative symptoms (37.1%) compared to patients without negative symptoms (25,5%, p = 0.011) and to healthy controls (22.8%, p = 0.015). As Theory-of-Mind (ToM) deficits [31] are a hallmark of most schizophrenic spectrum phenotypes and have shown to be particularly severe in patients with deficit schizophrenia [46], those results may add plausibility to the association of the C393T T-allele with lower cognitive empathy and perspective taking found here in our study.

### Increased empathy scores in T-allele carriers

The fact that C393T genotype was negatively correlated with CES and PT scores revealed an inverse effect of this genotype compared to the ordering of genotype we had hypothesized based on previous research. On the contrary, the C-allele that had been shown to constitute a risk factor in medical conditions such as hypertension [47,48] and metabolic conditions [27], was correlated with higher scores of dispositional cognitive empathy in female participants.

Although the etiology of a possibly lower cognitive empathy in T allele carriers is largely elusive, the T-allele may be associated with a lower responsiveness of the autonomous nervous system (ANS) compared to the C-allele [49,50]. Of note, the ASN seems to be critically involved in the activation of both emotional and cognitive empathic responses [25,51] by providing embodied simulation on the basis of visceromotor representations of other persons’ experience [11] and thus also producing information for higher-order ToM processes. Interestingly, Oterino et al. [49] found that the C393C- allele is associated with higher sympathetic nervous system reactivity in a sample of 365 migraine patients and 347 healthy controls, presumably due to an increased alpha subunit activation leading to enhanced coupling of D1-like dopamine receptors to adenylyl cyclase. Likewise, Yasuda et al. [50] found that T-allele carriers showed a significantly smaller high-frequency component in power spectral analysis of heart rate variability in supine rest compared to CC homozygotes in a sample of 137 young, healthy Japanese males. Autonomous reactions to orthostasis such as an increase in heart rate and sympathetic responsiveness were significantly lower in T-allele carriers compared to CC carriers. Conversely, the increased sympathetic responsiveness of CC homozygotes that has also been linked with the pathogenesis of hypertension [48], might provide an explanatory model for the stronger empathic reaction observed in our study. This explanation, however, is hypothetical at the moment and needs further research on the interactions of C393T genotype, ANS activity and psychometrical empathy scores.

### Specific effect of T393 genotype on cognitive empathy

Although these findings supply a preliminary explanation for a possibly increased empathic disposition in C-allele carriers, they fail to clarify why we observed a specific genotype effect in the cognitive, but not in the emotional domain in our sample. To resolve this issue, genetic imaging studies would be needed to explore possible neuroplastic effects of the C393T genotype on brain structures such as the cingulo-insular circuit involved in emotional empathy and the prefrontal-temporo-parietal circuit involved in ToM processes [11]. In the above-mentioned study, Minoretti et al. [45] imply such a plastic effect of the C393T polymorphism. They hypothesize that the TT genotype might be associated with deficit schizophrenia due to the fact, that higher levels of Gαs expression lead to an increased activation of adenylyl cyclase, resulting in cAMP accumulation and a consecutive promotion of proapoptotic processes. Although in healthy populations, endophenotype differences can be expected to be less marked, genotype effects on local gray matter density and functional connectivity with an impact on empathy are being reported in an increasing number of studies [52–54]. The endophenotype approach, which has previously been discussed in the context of cooperative social dispositions [9], could therefore be a promising direction of research concerning the effects of variations in G-protein encoding genes.

### Sex-specific effects of the C393T genotype

Another unresolved issue is the sex-specific association of the C393T polymorphism with IRI empathy scores observed in our study. The fact that the male sample of 231 participants achieved more statistical power for detecting a possible association of genotype with empathy than the female sample of 190 participants (88% vs. 81%) adds plausibility to the assumption that the association might be considerably weaker or absent in males. Notably, the absence of significant associations of genotype with the cognitive empathy scores in the complete sample (CES: p = 0.055 and PT: p = 0.057) might, therefore, be due to the excess of males over females. A tentative Jonchkeere-Terpstra analysis of a sample containing the 190 female and 190 randomly drawn male participants resulted in a significant association of both scores (CES: p = 0.017 and PT: p = 0.030) based on 10,000 Monte Carlo simulations, while all the other scores remained far from significant. Considering the apparent importance of sex differences, equal proportions of male and female participants might be advisable in future studies.

A markedly sex-specific effect of this polymorphism has been observed by Bachmann et al. [55] in a study on aseptic loosening after total hip arthroplasty, with the TT genotype posing a protective factor in women, but a risk factor in men. Although these results show that the polymorphism can have sexually dimorphic effects, the underlying mechanisms remain unclear.

However, two hypotheses may be worth of consideration in future research:

1. Gene-by-environment interactions: As is the case in other empathy-related genetic variations, the effects of the C393T polymorphism on dispositional empathy could also be subject to gene-by-environment interactions. For example, OXTR polymorphisms have been shown to induce a differential susceptibility to the social environment, thus leading to a marked influence of social experience on the development of empathic traits [52]. This differential social susceptibility [56] might also modulate the formative influence of gender-specific life strategies and role models in phylogenetic and ontogenetic development. Additional psychometry like the BEM Sex Role Inventory [57] could be applied to control for the influence of individual socialization.
2. Indirect gene-by-gene interactions: Variations of the GNAS gene, which have been robustly linked with the regulation of gonadal steroid levels by means of adenylyl cyclase activation [58], might indirectly modulate the activity of other empathy-related genes, whose activation seems to be partly hormone-dependent. Especially the OXT gene, which encodes the oxytocin precursor protein oxytocin/neurophysin I prepropeptide, contains an estrogen response element (EREs) at approximately -160 nucleotids upstream from the transcription start site of the OXT gene, resulting in a stimulation of the OXT promoter by estradiol [58].

Further research into possible gene-by-environment and gene-by-gene interactions concerning the C393T GNAS polymorphism is required to clarify underlying effective mechanisms.

### Strengths and limitations of the study

As the *GNAS* C393T T allele frequencies show considerable ethnic differences, ranging from 75% in African to 46% in Caucasian subjects [59], the results based on our sample of 421 Caucasian blood donors cannot necessarily be generalized to other populations. Due to the fact that cultural differences have been shown to influence the associations between polymorphisms and social preferences [60], cultural background can be another confounding factor. Although IRI empathy scores were not higher in our sample compared to the normative test samples [7,8] and donors receive remuneration, we are aware of the fact, that they might not be representative of the general population in terms of empathic disposition. Likewise, the exclusion of persons with specific lifestyle choices form blood donation as stipulated by German regulations may introduce another bias.

On the other hand, sample size (n = 421), life-time representativeness (18 to 74 years) and the presumably higher diversity among subjects recruited from a publicly accessible blood donor service compared to the common samples of university students can be considered to increase the representativeness of the data.

In our statistical analysis, we chose an extremely conservative approach towards controlling for multiple comparisons, which is a potential strength and a potential weakness at the same time. While it is indisputably appropriate to correct for testing more than one polymorphism in the same sample and for post-hoc testing of subgroups in the absence of a significant result in the complete sample, controlling for the subscales of a multidimensional psychometric test might be considered to overstep the mark in terms of avoiding type I error at the expense of the statistical power to detect true effects. A balanced approach towards significance correction is essential in the context of genetic association studies with empathy and other psychological constructs which are characterized by a polygenetic determination and by a marked gene by environment interactions, resulting in small effects of single genetic variations. In the case of the IRI, however, controlling for associations with subscales might nevertheless be advisable. As the subscales of the IRI only show small to moderate interscale-correlations, the developer of the instrument assumed “that the four qualities tapped by the IRI are indeed separate constructs, each related in specific and specifiable ways with other psychological measures” [8]. Since the IRI is one of the most frequently used questionnaires in empathy research and the present study is, to our knowledge, the first study controlling for associations with subscales, we invite further discussion about the proportionality of such a markedly conservative approach.

### Effect sizes compared to other polymorphisms

We reported an effect size of *GNAS* C393T genotype of r = -.209 (p = 0.004) on perspective taking (PT) for female participants, accounting for 4.37% of the variability in this cognitive empathy score. Although this effect is small by statistical standards, it is considerable in the context of the well documented polygenetic and environmental influences on the development of empathic dispositions. In particular, the C393T effect found in the present study clearly exceeds most of the effects found for the *OXTR* rs53576 polymorphism, the most extensively studied genetic variation in the context of empathy and sociality. In our recent study [13] for example, we reported an effect size of the *OXTR* rs53576 polymorphism on empathic concern (EC) in women of r = .146 (p = 0.04), accounting for 2.13% of variability in these scores. Naturally, even smaller correlations are found when associations of genotype are not restricted to measures of empathy, but are examined in relation to a wider concept of social disposition. In a meta-analysis of 48 studies associating the *OXTR* rs53576 polymorphism with different aspects of social functioning, including personality, social behaviour, psychopathy and autism (n = 17 559), Bakernans-Kranenburg [12] found a combined effect size of r = .02 (p = 0.07). Similarly, Li et al. [14] found an effect size of r = 0.02 (converted from Cohen’s d = 0.11, p = 0.02 according to McGrath & Meyer [61]) for associations with general sociality.

The effect reported here suggests, that the *GNAS* C393T genotype could be a new promising candidate for research on genetic influences on empathy.

## Conclusions

To the best of our knowledge, we present the first study examining associations of a gene encoding a G protein subunit with dispositional empathy. Our results suggest an association of *GNAS* C393T genotype with the cognitive empathic dimension of perspective taking in women, with higher scores in C compared to T allele carriers. Further studies are needed to examine replicability and to extend our understanding of the underlying mechanisms of signal transduction in empathy related circuits of the nervous system and of possible sexual dimorphisms.

## Supporting information

S1 File Questionnaire. German IRI questionnaire used in this study.
(PDF)

S2 File Study data. Spreadsheet containing all individual data, calculated IRI scores and a legend.
(XLSX)
